# Knotless Suture Staple Remplissage for Hill‐Sachs Lesions in the Beach Chair Position

**DOI:** 10.1002/atn2.70058

**Published:** 2026-04-29

**Authors:** Joseph M. Sliepka, Benjamin Lurie, Alan L. Zhang

**Affiliations:** ^1^ Department of Orthopaedic Surgery University of California‐San Francisco San Francisco California U.S.A.

## Abstract

The addition of remplissage to arthroscopic Bankart repair in the presence of a Hill‐Sachs lesion can help decrease recurrence of anterior shoulder instability. The remplissage procedure often involves passage of suture from anchors in the Hill‐Sachs lesion to the extra‐articular side of the shoulder capsule followed by knot tying. Extra‐capsular knot tying is difficult to visualize arthroscopically leading to potentially loose knot stacks. The use of knotless fixation in the proper configuration can eliminate the added complexity of this step when securing the infraspinatus tendon and capsule to the Hill‐Sachs lesion while increasing surgical efficiency. A dual knotless suture staple remplissage technique with all‐suture anchors in the beach chair position may provide an efficient and reproducible method for adding stability to anterior labral repair.

## Graphical abstract

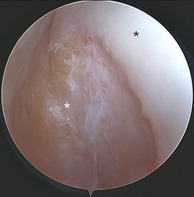

**VIDEO 1:** This video will describe our technique for performing an arthroscopic remplissage utilizing a knotless suture staple technique for Hill‐Sachs defects in the beach chair position. The patient is placed in the beach chair position and diagnostic arthroscopy is performed. Viewing from the anterior portal with a 30° arthroscopye the Hill‐Sachs lesion can be seen. This technique uses two 2.3 mm Iconix knotless all‐suture anchors (Stryker, Kalamazoo MI). We place the anchors for the remplissage prior to repairing the labrum because once the labrum is repaired, the humeral head sits more posteriorly and directly abuts the posterior capsule, making anchor insertion more difficult. A cannula is inserted into the posterior portal. The cannula is inserted all the way into the joint and then withdrawn until it is outside the infraspinatus tendon but still deep to the deltoid. The Iconix 2.3 mm anchor drill guide with the sharp T‐handle punch is used to pierce through the infraspinatus tendon and capsule in line with the Hill‐Sachs defect. The T‐handle punch is sharper than the standard obturator used with the Iconix and facilitates piercing the capsular tissue. The pilot hole for the anchor is then drilled and the anchor is malleted into place. Prior to placing the second anchor, the orange cannula is removed and reinserted into the shoulder after the sutures from the anchor are moved outside of it. This prevents the sutures from the 2 anchors from becoming tangled during insertion of the subsequent anchor. The drill guide and obturator are again used to penetrate the infraspinatus tendon, this time more inferiorly. The tissue between these 2 anchors will ultimately be compressed into the Hill‐Sachs defect at the end of the case. Now that both suture anchors have been placed through distinct parts of the infraspinatus, the posterior cannula can be removed and the suture snapped for later tightening. After the remplissage anchors are placed, attention is directed to the anterior labral repair. The labrum is elevated, anchors are placed, sutures are passed through the capsule and the labrum with a lasso. Knots are then tied away from the glenoid. Switching sticks are then used to place the camera back through the high interior portal to complete the remplissage procedure. The repair suture from 1 anchor is then passed through the looped shuttle suture from the other anchor. This shuttles the repair strand through the knotless mechanism of the opposing anchor. The repair strand is the tightened until a positive stop. This process is then repeated for the second anchor's repair suture to be docked by the first anchor's shuttle strand. No posterior cannula is needed for this step since both sutures were placed through the same defect in the deltoid. The repair strand for the second suture can then be tensioned to a positive stop to fully compress the infraspinatus tendon into the Hill‐Sachs defect. The two residual repair stitches are then cut flush. The Hill‐Sachs defect is no longer visible posteriorly when viewed from the anterior portal. Video content can be viewed at https://doi.org/10.1002/atn2.70058.

Anterior shoulder instability is among the most common shoulder pathologies seen by sports medicine surgeons, with an estimated incidence of 23 to 25 of 100,000 person‐years.^1^, ^2^ Risk factors for shoulder instability include younger age, male sex, participation in contact sports, and hyperlaxity.^3^, ^4^, ^5^

In the presence of a Hill‐Sachs lesion, isolated Bankart repair leads to high recurrent instability rates, especially in the case of an off track lesion.^6^, ^7^, ^8^, ^9^, ^10^ Burkhart and De Beer helped define the importance of bone loss both on the glenoid and humeral sides by introducing the concept of glenoid track.^11^ This led to the remplissage procedure, where tissue from the infraspinatus tendon and posterior capsule are used to fill the defect of the Hill‐Sachs lesion. Purchase et al.^12^ first described the remplissage, which is typically done as an adjunct to arthroscopic labral repair in the setting of concomitant Hill‐Sachs lesion. Outcomes after arthroscopic Bankart repair with remplissage have been encouraging, with lower recurrence rates near 5% to 10%.^7^, ^8^, ^9^, ^10^ Currently, there are no clearly defined indications for remplissage, however, those that have been proposed include off track or engaging Hill‐Sachs lesions, including in the setting of subcritical bone loss.^8^, ^13^, ^14^, ^15^

The primary complication with remplissage is impairment of postoperative external rotation, however evidence is conflicting.^13^, ^16^, ^17^, ^18^, ^19^ Another challenge associated with remplissage is tying down the construct with limited visualization. As described by Purchase, the remplissage is typically done before the Bankart repair and capsular space is closed down.^12^ However, visualization from the anterior portal of the Hill‐Sachs lesion can be difficult, and knots placed extracapsularly cannot be seen tied down. This often requires the construct to be fixed “blind” while using tactile feedback. We describe our preferred remplissage technique for arthroscopic remplissage in the beach chair position utilizing 2 knotless anchors to create a suture staple construction for filling the Hill‐Sachs lesion without the need for knot tying.

## SURGICAL TECHNIQUE

**FIGURE 1:**
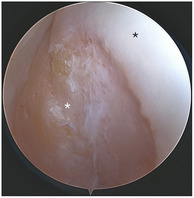
Intraoperative arthroscopic photo during diagnostic arthroscopy of the left shoulder in beach chair position viewing from a standard posterior portal with a 30° arthroscope shows a large Hill‐Sachs lesion over the posterior superior humeral head. The black and white asterisks represent humeral head cartilage and the Hill‐Sachs lesion, respectively.

The patient is positioned in the beach chair position, with the forearm secured with an arm positioner (AssistArm; Conmed, Largo FL) (Video 1). A posterior portal is made 1 cm lateral to the standard “soft spot”, followed by diagnostic arthroscopy to identify the Bankart and Hill‐Sachs lesions (Figure 1). Two anterior portals are then made in the rotator interval, one superomedial to assist with labrum elevation and suture passage, and the other inferolateral for anchor placement. The remplissage anchor placement is done after anterior portal placement and diagnostic arthroscopy, but before the arthroscopic Bankart repair. If the remplissage procedure is performed after the Bankart repair, the humeral head is shifted posteriorly against the capsule which makes visualization of the Hill‐Sachs lesion and anchor placement more difficult.

**FIGURE 2:**
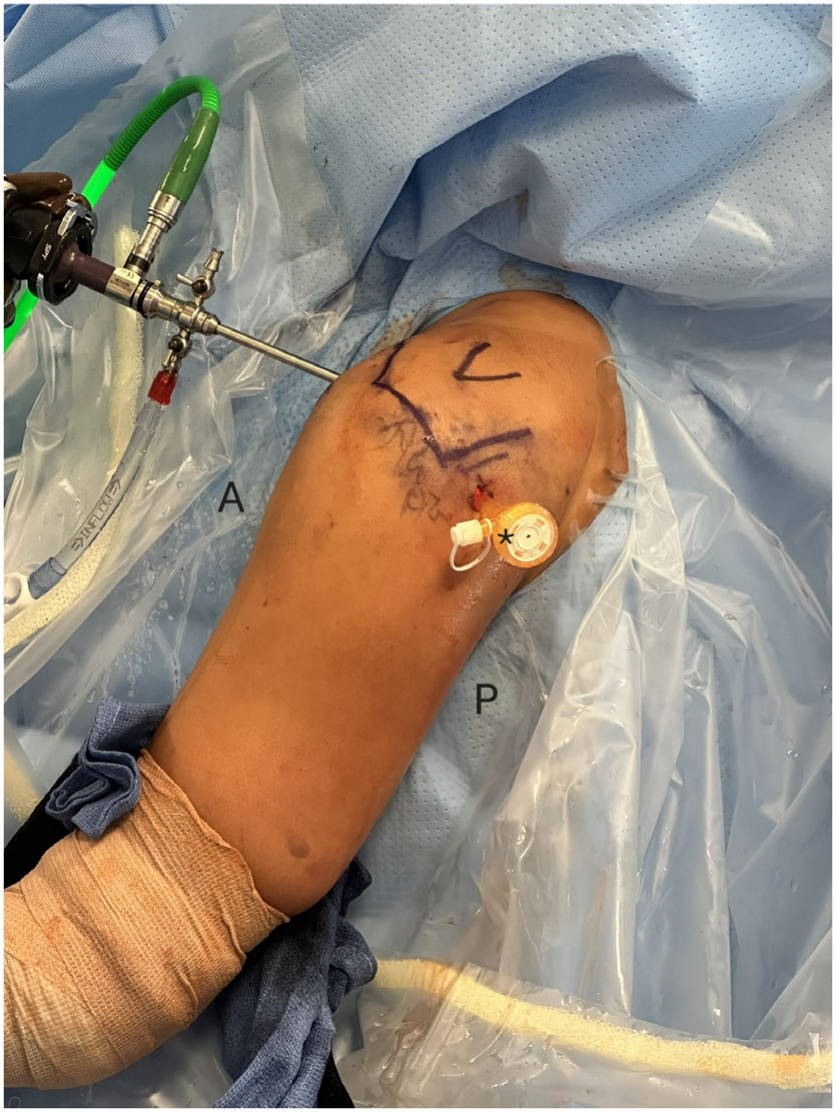
Intraoperative external photo of the left shoulder in beach chair position shows the arthroscope viewing from the anterior portal and a 5 mm cannula in the posterior portal as our standard positioning for remplissage procedure. The black asterisk represents the posterior portal. The letters A and P represent anterior and posterior, respectively.

**FIGURE 3:**
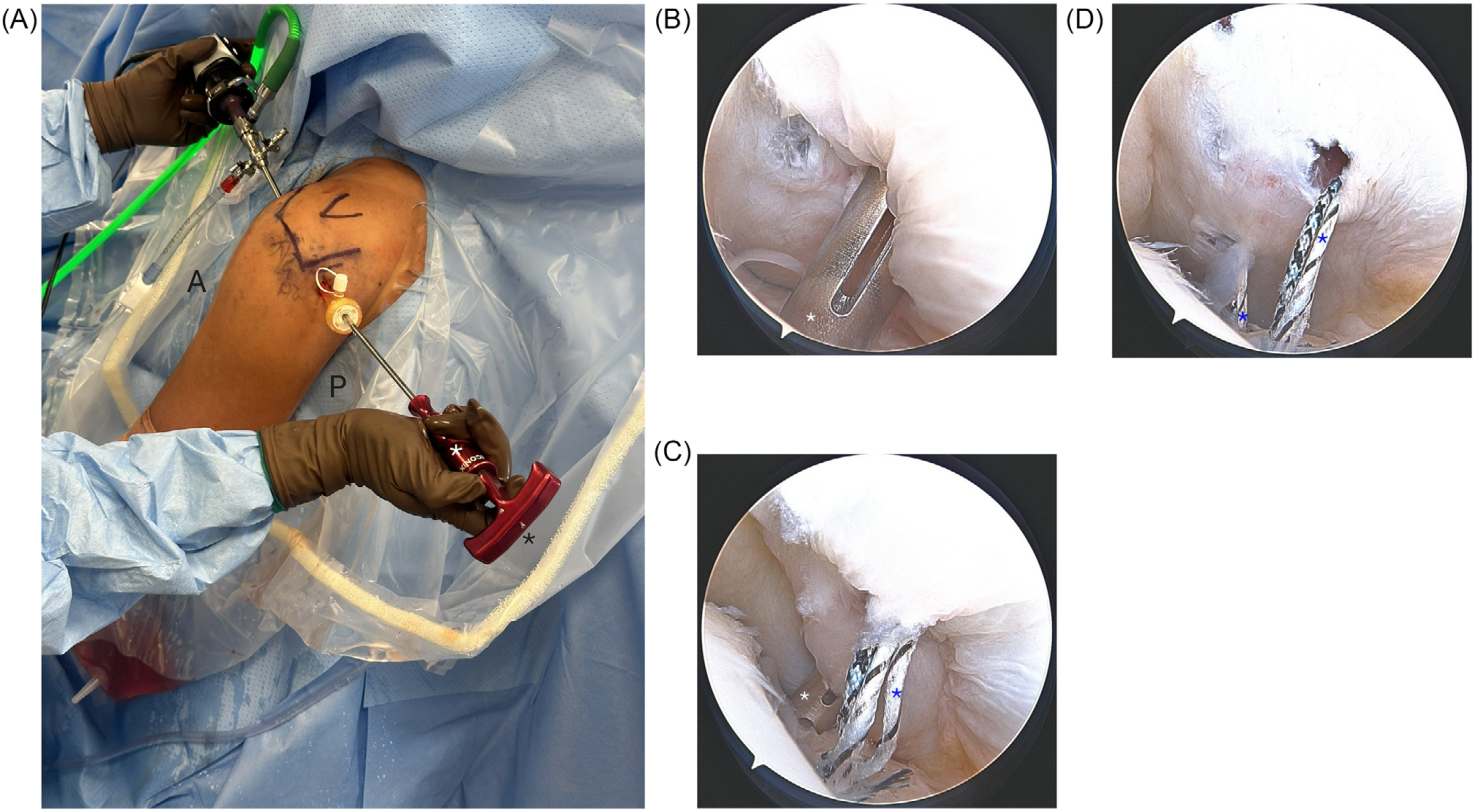
Intraoperative external and arthroscopic photos of the left shoulder in beach chair position viewing from the anterior portal with a 30° arthroscope. (A) With the arthroscope in the anterior portal, the anchor cannula and obturator can be placed through the posterior portal cannula. (B) The obturator is stopped short of the infraspinatus tendon and capsule and redirected inferior to the posterior capsulotomy to pierce the capsule and is placed into the inferior aspect of the Hill‐Sachs lesion. The anchor is then placed. (C) The process is repeated for the second anchor, this time redirected superior to the posterior capsulotomy and placed in the superior aspect of the Hill‐Sachs lesion. (D) The anchor cannula is then removed, leaving both sets of anchor sutures entering the joint adjacent to the posterior portal capsulotomy but exiting the skin through the same posterior portal incision. The black asterisk represents the sharp obturator within the anchor cannula used to penetrate the capsule. The white and blue asterisks represent the anchor cannula and sutures, respectively. The letters A and P represent anterior and posterior, respectively.

**FIGURE 4:**
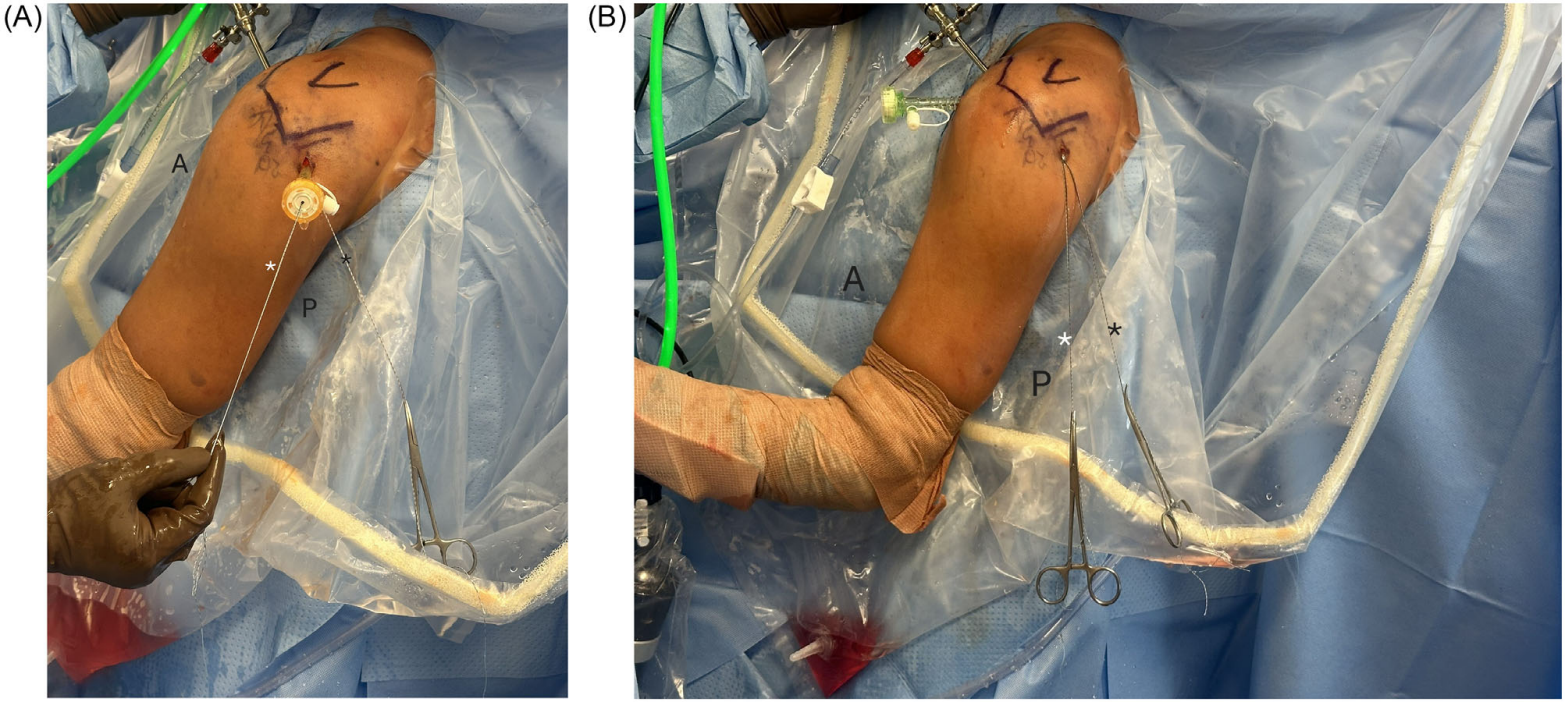
Intraoperative external photo of the left shoulder in beach chair position after anchor placement shows (A) two sets of anchor sutures coming out of the posterior portal, one through the cannula and the other outside the cannula, to help with suture management. (B) After the placement of both anchors, the posterior cannula can be removed. Both sets of anchor sutures are exiting the posterior portal incision and snapped independently to avoid mixing sutures. The black and white asterisks represent the sutures of the first (moved outside the cannula) and second (within the cannula) anchor, respectively. The letters A and P represent anterior and posterior, respectively.

After diagnostic arthroscopy, the camera is placed in the superomedial anterior portal with the aid of 2 switching sticks. A 5 mm diameter disposable cannula is placed in the posterior portal (Smith & Nephew, Andover, MA) (Figure 2). Under direct visualization, two 2.3 mm knotless all‐suture anchors (Iconix Knotless; Stryker, Kalamazoo, MI) are placed in the Hill‐Sachs lesion, one inferior and one superior. The disposable cannula is first withdrawn to the extra‐capsular space. Then the anchor drill guide loaded with a T‐handle punch is placed in the disposable cannula. Prior to entering the capsule, the T‐handle punch is used to redirect the anchor drill guide and pierce the capsule inferiorly in a separate location from the original posterior portal track. The T‐handle punch is then removed as the anchor drill guide is placed in the center of the Hill‐Sachs lesion. A 2.3 mm drill is then used to drill a tunnel to a depth of 21.5 mm after which the knotless all‐suture anchor is placed in the Hill‐Sachs lesion. The anchor contains one repair strand and one looped shuttle strand. The suture strands are pulled upwards slightly to gently set the anchor. To assist with suture management, after the first anchor is placed, the 5 mm disposable cannula can be removed and then replaced in the same track through a switching stick such that the sutures from the first anchor are now outside the cannula and clamped. The second anchor is then placed in the same fashion, this time with the T‐handle punch redirecting superiorly when piercing the capsule to place an anchor in the superior portion of the Hill‐Sachs lesion (Figure 3A‐D). The sutures from each anchor are clamped independently to avoid mixing sutures (Figure 4A and B). After both anchors are placed, switching sticks are utilized to place the camera back into the posterior portal/capsule and the surgeon can then proceed with the arthroscopic anterior labral repair.

**FIGURE 5:**
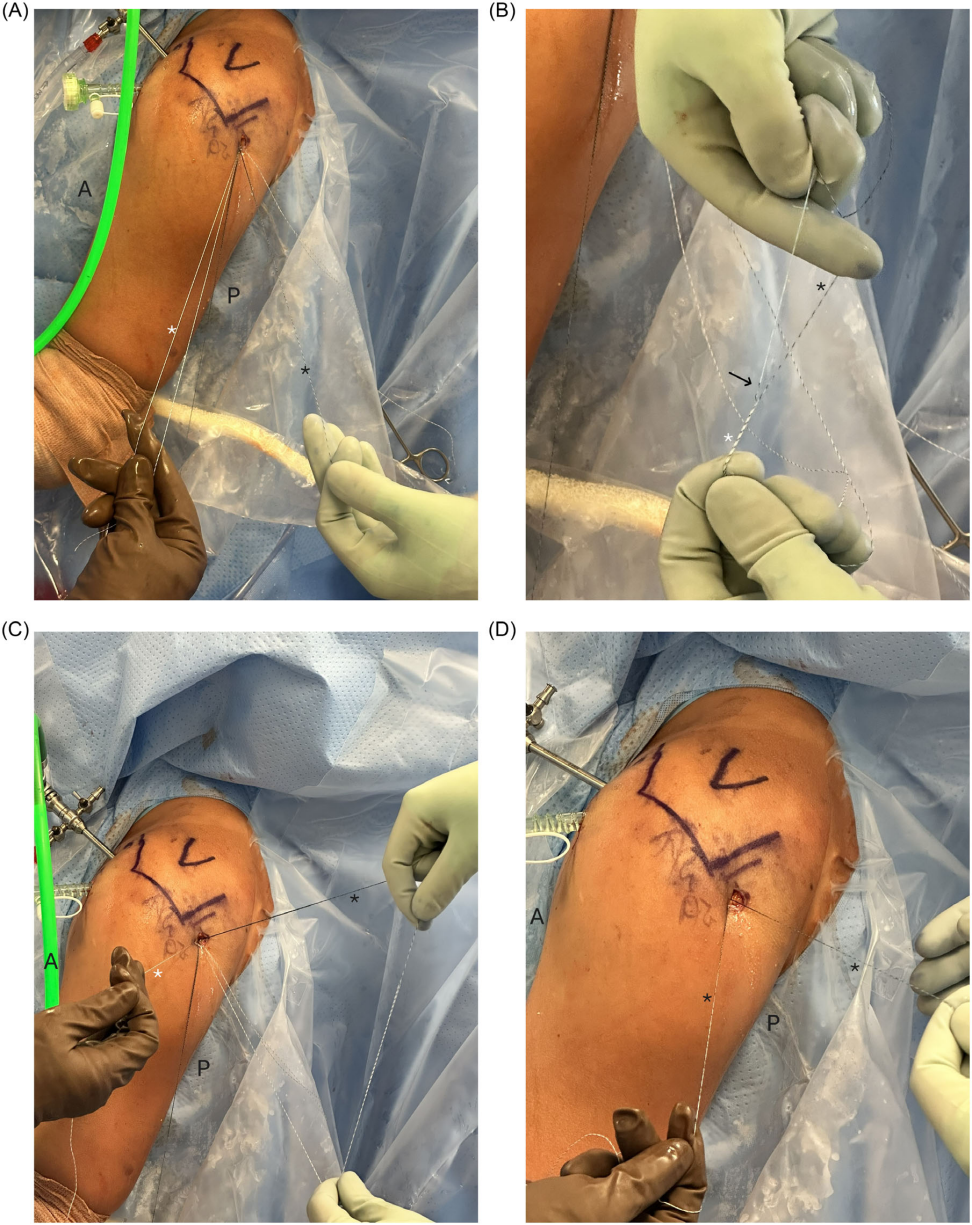
Intraoperative external photos of the left shoulder in beach chair position show tensioning of the construct. Of note, the posterior cannula has been removed. (A) The looped shuttle strand from one anchor and the repair strand from the opposite anchor are isolated. (B) The repair strand from one anchor is then passed through the looped shuttle suture from the other anchor. In this system, transition markings on the repair stitch (from solid to striped) demarcate the appropriate distance to overlap the repair stitch through the loop to ensure safe passage. (C) As the repair strand from the first anchor is passed into the opposite anchor by a pulsating upward force on the shuttling strand, the remaining sutures are held away with slight tension to avoid entrapment. (D) The repair strand from the second anchor is now passed into the first anchor by the shuttling strand of the first anchor and tightened. The black and white asterisks represent the repair and shuttle strands, respectively. The arrow is pointing to the transition point on the repair strand from solid to stripe demarcating the appropriate point at which to overlap the repair strand around the loop shuttle strand. The letters A and P represent anterior and posterior, respectively.

**FIGURE 6:**
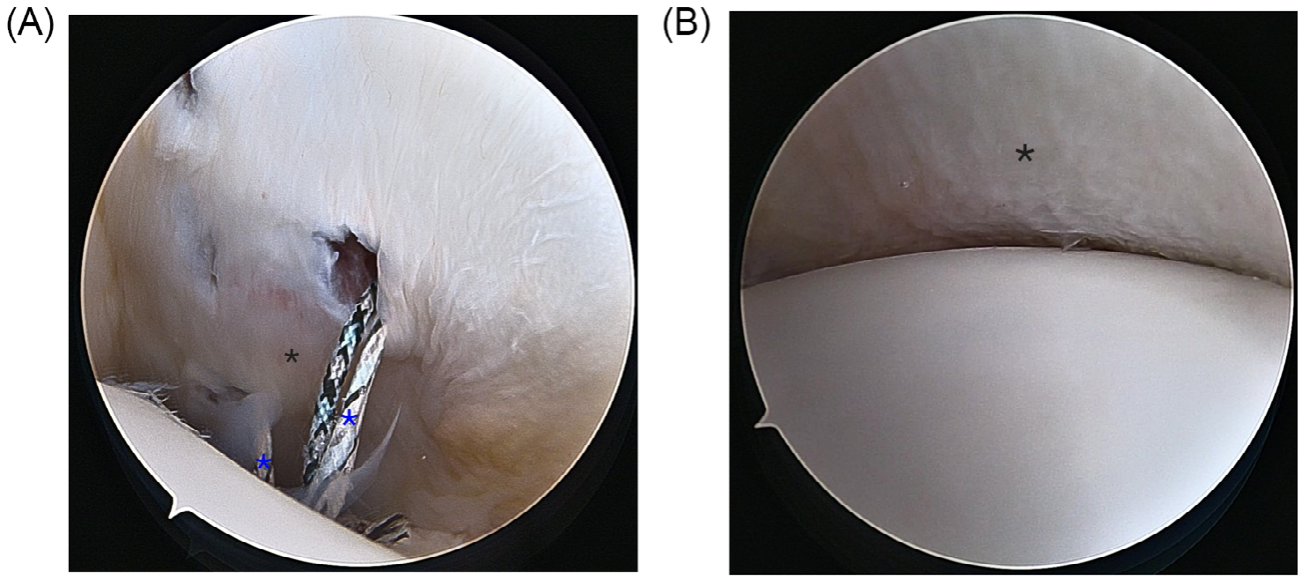
Intraoperative arthroscopic photos of the left shoulder in beach chair position viewing from the anterior portal with a 30° arthroscope. (A) This represents the view of the posterior shoulder after remplissage anchor placement but before tensioning. (B) This represents the view of the posterior shoulder after the repair strands have been looped through each other and then tensioned down. The capsular space behind the Hill‐Sachs lesion has been effectively closed. The blue asterisks represent the anchor sutures placed into the Hill‐Sachs lesion. The black asterisk represents the posterior capsule being tightened over the lesion.

**FIGURE 7:**
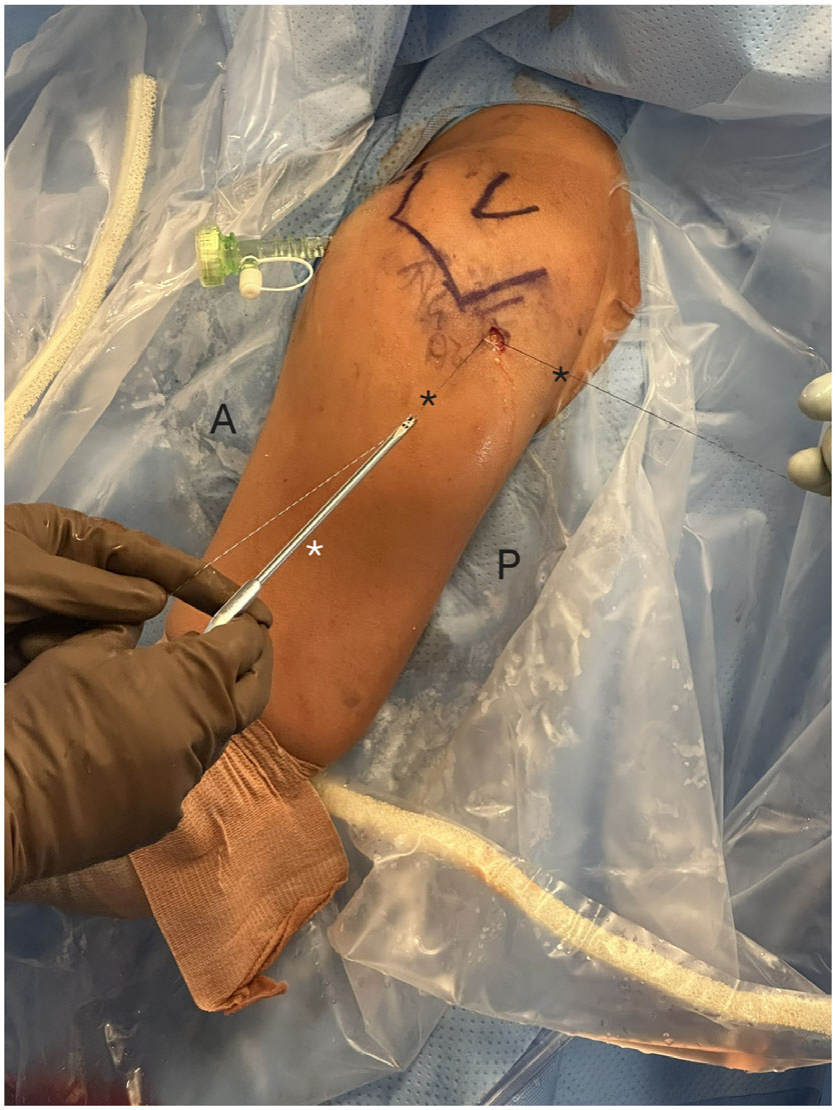
Intraoperative external photo of the left shoulder in beach chair position shows usage of an arthroscopic flush cutter being passed down the remplissage repair stitch through the posterior incision. Sutures are cut after both anchors are fully tensioned, completing the knotless technique. The black and white asterisks represent the repair sutures and the flush cutter, respectively. The letters A and P represent anterior and posterior, respectively.

Once the anterior stabilization is completed, the surgeon then completes the remplissage procedure. Switching sticks are again used to bring the camera back through the superomedial anterior portal. The sutures from each knotless anchor are then carefully separated. The repair strand from one anchor is then passed through the looped strand from the other anchor and pulled through, and this process is repeated for the other repair and looped sutures. In this step, care is taken to pass the repair strand through the loop of the shuttle strand until it reaches the demarcation of suture color change. At the demarcation, the repair strand is folded and pinched to the shuttle strand loop. The shuttle strand can then be pulled, passing the repair strand through the capsule and infraspinatus tendon. The shuttle strand used to pass the repair strand can then be discarded (Figure 5A‐D). This effectively loops each repair strand through the infraspinatus tendon across the posterior capsulotomy. The tail of each repair strand is then pulled in a short pulsating fashion until there is a positive stop, reducing the infraspinatus and capsule into the Hill‐Sachs lesion without the need for knot tying (Figure 6A and B). The final construct with posterior capsule filling the Hill‐Sachs lesion can be visualized from the anterior portal. As these are knotless anchors, the residual repair stitches can then be cut flush (Figure 7).

### Postoperative Rehabilitation

The addition of our remplissage does not change our postoperative shoulder instability protocol. Patients remain in a sling for a total of 4 weeks postoperatively, during which time they can work on wrist and finger range of motion. After 4 weeks, they can discontinue sling use to begin range of motion exercises. Strengthening begins at 3 months, followed by return to sports at 6 months after surgery.

## DISCUSSION

The addition of remplissage to arthroscopic Bankart repair for shoulder instability in the presence of a Hill‐Sachs lesion has grown in popularity as evidence for its benefit becomes more apparent. There are many techniques to remplissage; however, those that involve knot tying present a challenge of unknown construct tension given lack of visual feedback when securing the tendon and capsule.

Our technique provides the advantage of allowing for knotless fixation. Our technique provides the advantage of allowing for knotless fixation (Table 1). By eliminating the need for knot tying, our knotless suture staple technique may simplify the remplissage procedure, increasing efficiency. In addition, our procedure can be performed easily in the beach chair position for surgeons that prefer this set‐up instead of the lateral decubitus position. This technique also makes use of a sharp punch as the obturator within the anchor drill guide to pierce through the infraspinatus tendon and capsule. This technique also makes use of a sharp punch as the obturator within the anchor drill guide to pierce through the infraspinatus tendon and capsule (Table 2). While other techniques require a penetrator instrument to pierce through the capsule and pass suture through different locations in the capsule, our technique eliminates that extra step. Lastly, this technique can be done from a single posterior portal incision without the need for accessory portals or additional posterior incisions.

**TABLE 1:** Advantages and Disadvantages of Knotless Suture Staple Remplissage Technique

Advantages	Disadvantages
By using the anchor cannula to pierce through tendon and capsule, this eliminates the added step of using a penetrator to pass suture after anchor placement	Requires 2‐anchor use for all repairs, regardless of size of Hill‐Sachs lesion which may represent an added step compared with other techniques that only place one anchor for smaller Hill‐Sachs lesions
Tensioning requires simple pulling of each repair strand rather than use of a knot pusher	Two‐anchor use may increase OR cost due to implants
Once anchors are placed, the construct can be tensioned at any point in the procedure, before or after labral repair	If the knotless all‐suture anchor is not well seated in bone, it may have a risk of pull out as the strength of all‐suture anchors is lower than that of screw‐in type anchors
Knotless fixation without the need for tying suture can increase efficiency of surgery	
Knotless looped fixation eliminates the need for “blind” tensioning and knot tying	
This procedure can be performed solely through the use of the posterior portal and does not require additional posterior percutaneous incisions or portals	
This procedure can be performed in the beach chair position	

**TABLE 2:** Pearls and Pitfalls of Knotless Suture Staple Remplissage Technique

Pearls	Pitfalls
Placing the remplissage anchors prior to anterior labral repair can improve with visualization of Hill‐Sachs lesion	Waiting to place anchors until after the labral repair can limit visualization due to posterior shift humeral head making anchor placement more difficult
Use of a sharp T‐handled punch as the obturator for the anchor drill guide can facilitate passage through the infraspinatus and capsule prior to anchor placement	Percutaneous placement of the anchor through separate incisions instead of the same posterior working portal may introduce soft tissue bridges superficial to the infraspinatus tendon
After first anchor placement, removal and replacement of the posterior portal cannula such that the sutures of the first anchor are outside the cannula can help with suture management	As each anchor has 3 sutures and they are placed in the same posterior tract, lack of attention to each anchor's suture limbs can lead to sutures being mistakenly crossed and shuttled into the wrong anchor
Marking the suture limbs from each anchor as shuttling strands and repair strands is critical to secure a staple fixation	

The knotless suture staple technique also has disadvantages. Limitations of this technique include requiring the placement of two anchors to create the staple construct. In other techniques, one anchor may be all that is needed for a remplissage procedure. When compared with these cases, our technique would yield higher surgery cost due to increased implant utilization. In addition, if the knotless all‐suture anchor is not well seated in bone, it may have a risk of pull out as the strength of all‐suture anchors is lower than that of screw‐in type anchors.

In the presence of a Hill‐Sachs lesion, remplissage helps reduce recurrence rates of anterior shoulder instability. Performing remplissage utilizing a dual knotless suture staple technique provides a safe and efficient way to augment anterior stabilization.

## DISCLOSURES

The authors declare the following financial interests/personal relationships which may be considered as potential competing interests: A.L.Z. reports a relationship with Stryker that includes: consulting or advisory; reports a relationship with DePuy Synthes Mitek Sports Medicine that includes: consulting or advisory; reports a relationship with CONMED Corporation that includes: consulting or advisory; reports a relationship with *Arthroscopy* that includes: Associate Editor. The other authors (J.M.S., B.L.) declare that they have no known competing financial interests or personal relationships that could have appeared to influence the work reported in this paper.
